# Outer membrane protein a of *Salmonella enterica *serovar Typhimurium activates dendritic cells and enhances Th1 polarization

**DOI:** 10.1186/1471-2180-10-263

**Published:** 2010-10-15

**Authors:** Jun Sik Lee, In Duk Jung, Chang-Min Lee, Jin Wook Park, Sung Hak Chun, Soo Kyung Jeong, Tae kwun Ha, Yong Kyoo Shin, Dae Jin Kim, Yeong-Min Park

**Affiliations:** 1 Department of Microbiology and Immunology, Albert Einstein College of Medicine, Bronx, NY 10461, USA; 2 Department of Microbiology and Immunology & National Research Laboratory of Dendritic Cell Differentiation & Regulation, Pusan National University School of Medicine, Yang-san 626-770, South Korea; 3 Department of Surgery, Busan Paik Hospital, Inje University, College of Medicine, Busan, 614-735, South Korea; 4 College of Medicine, Chung-Ang University, 221 Heukseok-Dong, Dongjak-Gu, Seoul, Korea; 5 Department of Anatomy, College of Medicine, Chung-Ang University, 221 Heukseok-Dong, Dongjak-Gu, Seoul, Korea

## Abstract

**Background:**

Typhoid, which is caused by *Salmonella enterica *serovar Typhimurium, remains a major health concern worldwide. Multidrug-resistant strains of *Salmonella *have emerged which exhibit increased survivability and virulence, thus leading to increased morbidity. However, little is known about the protective immune response against this microorganism. The outer membrane protein (Omp)A of bacteria plays an important role in pathogenesis.

**Results:**

We purified OmpA from *S. enterica *serovar Typhimurium (OmpA-sal) and characterized the role of OmpA-sal in promoting adaptive and innate immune responses. OmpA-sal functionally activated bone marrow-derived dendritic cells by augmenting expression of CD80, CD86, and major histocompatibility complex classes I and II. Interestingly, OmpA-sal induced production of interferon-γ from T cells in mixed lymphocyte reactions, thus indicating Th1-polarizing capacity. The expression of surface markers and cytokine production in dendritic cells was mediated by the TLR4 signaling pathway in a TLR4 Knock-out system.

**Conclusions:**

Our findings suggest that OmpA-sal modulates the adaptive immune responses to *S. enterica *serovar Typhimurium by activating dendritic cells and driving Th1 polarization, which are important properties to consider in the development of effective *S. enterica *serovar Typhimurium vaccines and immunotherapy adjuvant.

## Background

Dendritic cells (DCs) are professional antigen-presenting cells (APCs) that play key roles in the regulation of immune responses to a variety of antigens and immune sentinels as initiators of T cell responses against microbial pathogens [1-3]. In addition, during inflammation or infection, DCs are mobilized in and out of the peripheral tissues. Activated DCs are targeted to secondary lymphoid organs and toward T cell activation by antigen presentation [4,5]. DCs can capture degraded bacteria or protein of bacteria and present their antigens on major histocompatibility complex (MHC) class molecules to T cells [6]. As a result, an adaptive immune response that specifically targets bacteria-derived antigens is initiated. Maturing DCs then migrate to the lymphoid organs, where they activate naïve T cells by stimulating antigenic peptide-presenting MHC type I and II receptors and their co-stimulatory molecules [7]. Therefore, DCs provide a link between innate and adaptive immune responses.

*Salmonella *species cause typhoid fever and gastroenteritis in humans and pose a global threat to human health [8]. *Salmonella *also infect broad array of animals, resulting in diseases ranging from gastroenteritis to life-threatening systemic infections [9,10]. A recent report has shown that *Salmonella enterica *serovar Typhimurium is a bacterial pathogen capable of interfering with DC functions, and causes a typhoid-like disease in mice [11]. It has also been reported that the effect of selectively reduced intracellular proliferation of *S. enteria *serovar Typhimurium within APCs limits both antigen presentation and development of a rapid CD8 T cell response [12]. Outer membrane protein (Omp) from *S. enteria *serovar Typhimurium was shown to contribute to confers protection against typhod.

However, it is still not known if hosts mount protective immune responses against *S. enterica *serovar Typhimurium, thus understanding how the immune system responds to these bacteria is essential for the development of an effective *S. enterica *serovar Typhimurium vaccine.

In this study, we determined the effects of a non-cytotoxic concentration of purified outer membrane protein A from *S. enterica *serovar Typhimurium (OmpA-sal) on the maturation and function of DCs. Our findings suggest, for the first time, that exposure to OmpA-sal induces phenotypic and functional maturation of DCs. Interestingly, exposure to OmpA-sal induced the activation of ERK1/2 and p38 MAPK via TLR4. The findings presented herein suggest that OmpA-sal induces activation of DCs and initiates an adaptive immune response by polarizing T-cell development to a Th1 response, information which will prove crucial in the development of a *S. enterica *serovar Typhimurium vaccine.

## Results

### OmpA-sal induces DC maturation

**Figure 1:**
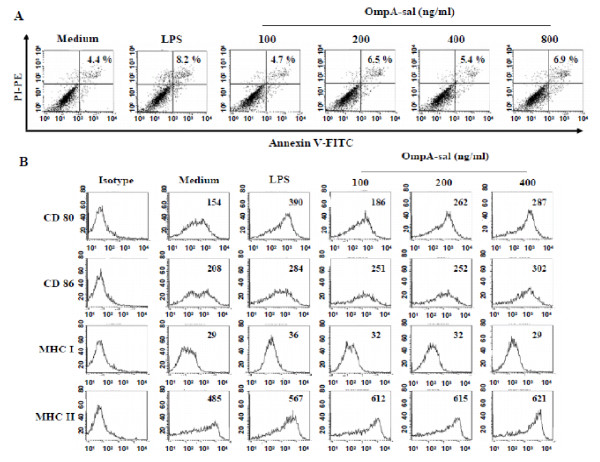
**OmpA-sal is not cytotoxic and induces the expression of co-stimulatory molecules in DCs**. BM-DCs were cultured for 24 h in the presence of 200 ng/ml of LPS or 100, 200, 400, and 800 ng/ml of OmpA-sal and analyzed by flow cytometry. The DCs were stained with annexin V and PI. The percentage of positive cells is indicated (A). The cells were gated to exclude CD11c^+ ^cells. Medium, untreated control; LPS, positive control. DCs were stained with anti-CD80, anti-CD86, anti-MHC class I, and anti-MHC class II molecules (B). The data are representative of three experiments that yielded similar results.

We purified OmpA-sal from *E. coli *and assessed its cytotoxicity on DCs because the purified OmpA-sal was derived from *S. enterica *serovar Typhimurim. DCs were treated with various concentrations of OmpA-sal for 24 h. There were no statistically significant differences in the percentages of dead cells in DC cultures exposed to as much as 800 ng/ml of OmpA-sal, the concentration at which cell death was detected by annexin V/PI staining (Fig. 1A). This indicated that our recombinant OmpA-sal was not cytotoxic to DCs and did not contain amounts of endotoxin that would interfere with our studies using concentrations < 400 ng/ml. To determine the effects of OmpA-sal on the maturation of sentinel DCs into effector DCs, BM-derived DCs were cultured with GM-CSF and IL-4 for 6 days under standard conditions, followed by 1 day in the presence of 100, 200, and 400 ng/ml of OmpA-sal. LPS was used as a positive control. The resulting populations of DCs were analyzed by flow cytometry for expression of co-stimulatory molecules involved in T cell activation. OmpA-sal-treated DCs had increased expression of DC maturation co-stimulatory markers (DC80, CD86, MHC class I, and MHC class II; Fig 1B). Interestingly, the expression of CD86 and MHC class II by OmpA-sal-treated DCs was higher than LPS-treated DCs. These results indicated that OmpA-sal induces DC maturation in a dose-dependent manner.

### OmpA-sal reduces the endocytic activity of DCs

**Figure 2:**
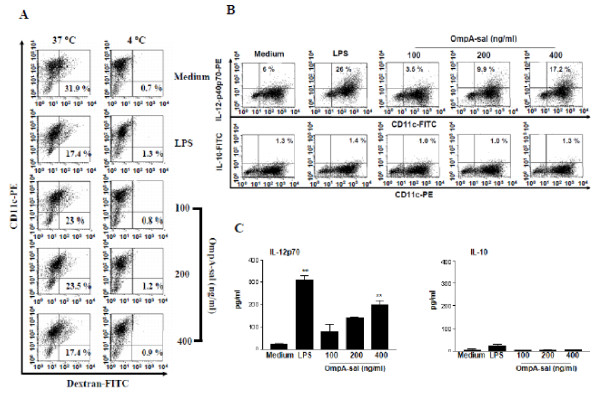
**Treatment of OmpA-sal decreases endocytic capacities of DCs and induces production of IL-12**. On day 6, the cells were cultured at standard conditions for another 24 h in the presence of 200 ng/ml of LPS or 100, 200, and 400 ng/ml of OmpA-sal and harvested, and stained with a PE-conjugated anti-CD11c^+ ^antibody. Endocytic capacity at 37°C or 4°C was assessed by dextran-FITC uptake (A). The percentage of positive cells is indicated for each condition and is representative of the data of three separate experiments (B). Analysis of IL-12p70 and IL-10 cytokine production in magnetic bead-purified DCs by ELISA (C). The data are the means and standard deviation of three experiments. *p < 0.05, **p < 0.01 vs. untreated DCs.

Immature DCs are efficient in the capture and endocytosis of antigens. These cells can internalize large amounts of antigen through each fluid-phase uptake via macropinocytosis and receptor-mediated uptake. However, in the case of mature DCs, the capacity to capture antigen and confer potent co-stimulatory activity for T cells is decreased [13]. We investigated whether OmpA-sal-treated DCs had reduced endocytic activity characteristic of functionally mature DCs. As shown in Fig. 2A, the percentage of double-positive cells was lower in the LPS-treated DCs than in the untreated DCs. Similarly, the percentage of double-positive cells was lower in the OmpA-sal-treated DCs compared with untreated DCs. These data show that the OmpA-sal-treated DCs had reduced endocytic activity, which indicates functional maturity.

### OmpA-sal increases the number of IL-12-producing DCs, but not IL-10

APC, such as DCs, have been shown to direct Th1 development by production of IL-12 [14]. The effector factors that drive the development of Th1- and Th2-type T cells are IL-12 from DCs and IFN-γ or IL-4 from T cells. We determined whether OmpA-sal induced differentiation of Th1 subsets, and IL-12-producing DCs were analyzed by flow cytometry and ELISA. We also investigated the production of both intracellular IL-12p40p70 and bioactive IL-12p70 in OmpA-sal-treated DCs. As shown in Fig. 2B, OmpA-sal treatment of DCs increased the percentage of IL-12-producing cells compared with the results obtained for untreated DCs. Next, we investigated the production of IL-10, a pleoiotropic cytokine known to have inhibitory effects on the accessory functions of DCs, which appears to play a role in Th2 immune responses. The production of IL-10 was detectable similar to that of negative controls (Fig. 2C).

### OmpA-sal-treated DCs enhances Th1 polarization and IFN-γ production

**Figure 3:**
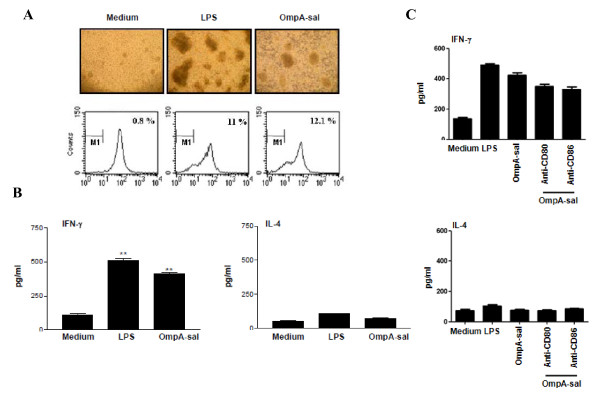
**OmpA-sal-treated DCs induces proliferation of allogenic T cells and enhanced Th1 resoponse *in vitro***. The DCs were incubated for 24 h in medium alone, in 200 ng/ml LPS, or in 400 ng/ml of OmpA-sal. The DC were washed and co-cultured with T cells. Cluster formation was assessed after 72 h. One representative experiment of three is also included in the figure, showing a representative field in a culture well photographed using an inverted phase contrast microscope and a mixed lymphocyte reaction was allowed to proceed for 3 days, T-cell proliferation was analyzed by flow cytometry and presented as a percentage of dividing cells (A). Cells were then examined for cytokine release after 48 h. IFN-γ and IL-4 were measured by ELISA in culture supernatants (B, C). Medium represents the chemically untreated control group. Similar results were obtained and expressed as the means (±SD) from four separate experiments. **p < 0.01 vs. untreated DCs.

To determine whether or not OmpA-sal-treated DCs stimulate CD4^+ ^T cell activation, we stimulated DCs with 400 ng/ml of OmpA-sal for 24h and performed an allogeneic mixed-lymphocyte reaction. CD4^+ ^splenic T cells from BALB/c mice were co-cultured with OmpA-sal-treated DCs derived from C57BL/6 mice. The OmpA-sal-treated DCs induced an advanced rate of T-cell proliferation compared to the untreated control DCs (Fig. 3A). In addition, we determined the cytokine production of CD4^+ ^T cells stimulated by OmpA-sal-treated DCs. As shown in Fig. 3B, allogeneic T cells primed with OmpA-sal-treated DCs produced a Th1 cytokine profile that included large amounts of IFN-γ and low amounts of IL-4. These data suggest that OmpA-sal enhances the immunostimulatory capacity of DCs to stimulated T cells. Moreover, we investigated whether cosignaling via CD80 and/or CD86 enhances Th1 response, we found that blockage of CD80 and CD86 decreased IFN-γ production. These data suggested that both CD80 and CD86 are essential for the Th1 response of OmpA-sal treated DCs.

### OmpA-sal induces DC maturation by TLR4 signaling

**Figure 4:**
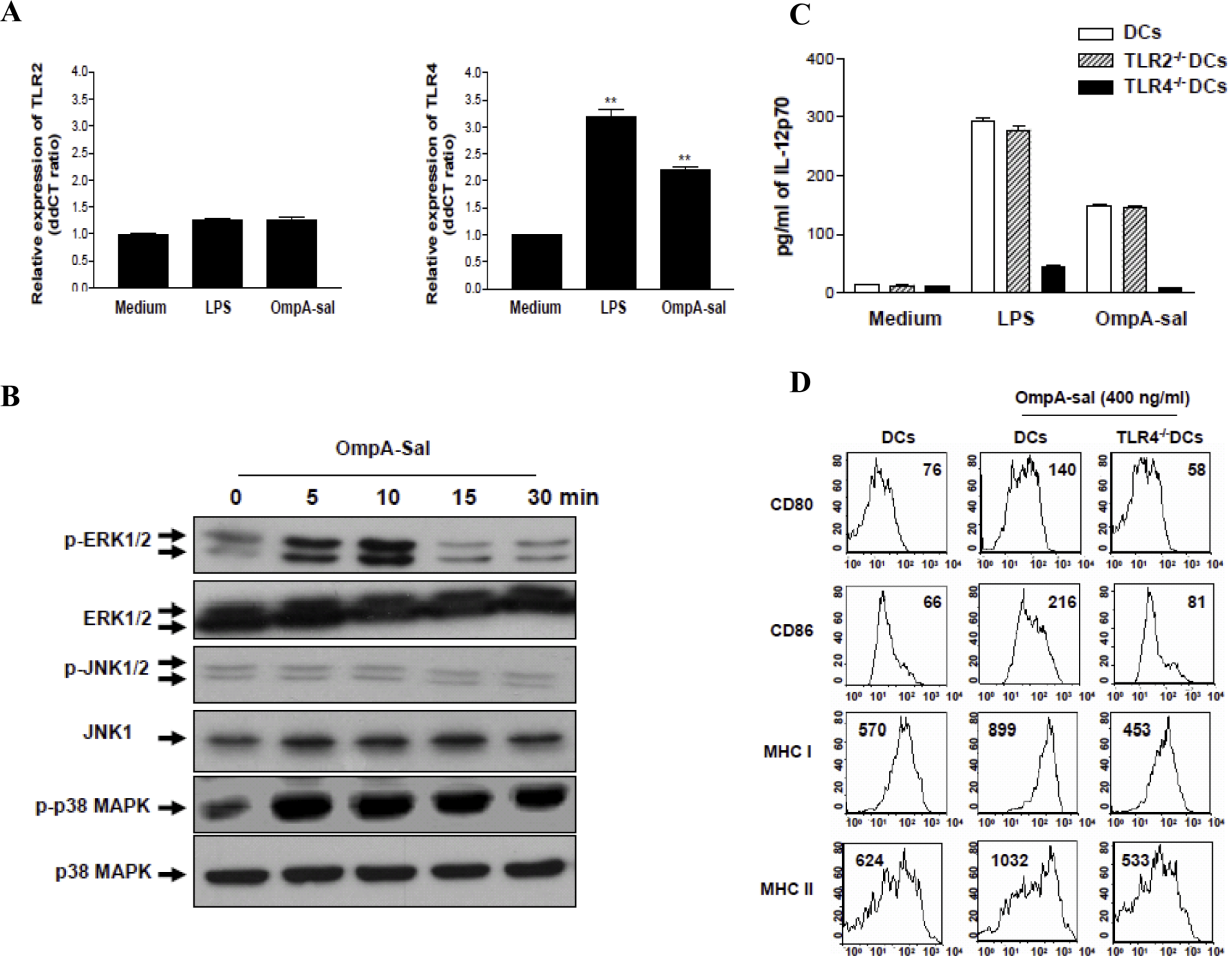
**OmpA-sal induces TLR4 expression, ERK activation, and p38 MAPK activation, but not JNK activation**. Total RNA was extracted, and quantitative real-time PCR was performed using sequence-specific primers for TLR2 and TLR4 (A).. Cell lysates were prepared and blotted with anti-phopho-p38, anti-p38, anti-phopho-ERK1/2, anti-ERK1/2, anti-phopho-JNK1/2, and anti-JNK1 antibody. A signal was detected with biotinylated goat-anti mouse IgG and visualized using enhanced chemiluminescence (B). DCs, TLR2^-/-^DCs, and TLR4^-/-^DCs were cultured for 24 h in the presence of 200 ng/ml of LPS or 400 ng/ml of OmpA-sal and the production levels of IL-12 analyzed by ELISA (C). BM-DCs and TLR4^-/-^DCs were cultured for 24 h in the presence of 400 ng/ml of OmpA-sal and surface markers analyzed by flow cytometry (D). The data are the means and standard deviation of three experiments. **, P < 0.01 for a compare with untreated DCs.

Toll-like receptors (TLRs) link innate and adaptive immune responses [15]. The DC response to TLR ligands depends on the activation of mitogen-activated protein kinases (MAPKs), including ERK1/2, JNK1/2, and p38 MAPK [16]. We determined the effects of OmpA-sal on TLRs and the MAPK signaling pathway. DCs were treated with 400 ng/ml of OmpA-sal and TLR activation was measured by real-time quantitative reverse transcription-PCR and phophorylation-specific Western blotting. The level of TLR4 mRNA was significantly higher in OmpA-sal-treated DCs than in untreated control DCs, but there was no change in TLR2 mRNA (Fig. 4A). Moreover, OmpA-sal enhanced the phosphorylation of ERK1/2 and p38 MAPK in DCs, but not JNK1/2 (Fig. 4B). To confirm whether or not the maturation of DCs by OmpA-sal was mediated by a TLR4-related signaling pathway, we isolated DCs from TLR2 and TLR4 knock-out mice, then measured IL-12 production in DCs by OmpA-sal treatment. The inducing effect of OmpA-sal on IL-12 production was completely inhibited by TLR4^-/- ^DCs, but it had no effect on TLR2^-/- ^DCs (Fig. 4C). Moreover, we demonstrated that OmpA-sal-treated TLR4^-/-^DCs had no increased expression of DC maturation co-stimulatory markers (DC80, CD86, MHC class I, and MHC class II; Fig 4D). These results indicate that the activation and maturation of DCs by OmpA-sal is involved in TLR4 signaling.

## Discussion

We have shown that OmpA-sal, a major virulence factor of *S. enterica *serovar Typhimurium, is a highly immunogenic protein that induces Th1 polarization of T cells by DC maturation. Some of the Omps from bacteria induce DC maturation and regulate Th1/Th2 immune responses [17-19]. Isibasi et al previously investigated the Omp of *Salmonella *as potential vaccine candidates, diagnostic antigens, and virulence factors [20]. However, the molecular mechanisms of the involvement of DCs and T cells in the immune responses still unknown. The lack of understanding of protective immunity against *S. enterica *serovar Typhimurium has hindered the development of an efficacious vaccine. In this study, we found that OmpA-sal induces activation and maturation of DCs, as demonstrated by the high expression of co-stimulatory and MHC class molecules on cell surfaces and reduced endocytic activity. In addition, OmpA-sal-treated DCs induced primary T cell stimulatory activity in an allogeneic mixed lymphocyte reaction and elicited Th1 polarization through high levels of IFN-γ and low levels of IL-4. We have also shown in the current study that various concentrations of OmpA-sal induce high expression of CD80, CD86, MHC class I, and MHC class II in DCs. Moreover, OmpA-sal-treated DCs produced high levels of IL-12, but not IL-10. These data suggest that OmpA-sal strongly induces activation and maturation of DCs, and as a result DCs transmit OmpA-sal to the adaptive immune response. Successful induction of an adaptive immune response is characterized based on which antigen is presented, the dose, and the duration of presentation [21-23]. In the case of antigen recognition, an intracellular/extracelluar signaling cascade leads to activation of APCs, which in turn promotes further activation of DCs and activated T cells, and results in proliferation of T cells and their differentiation into effector T cells [5]. Accordingly, T cell proliferation in mixed lymphocyte reactions is important for efficient induction of an adaptive immune response by interaction between DCs and T cells. In the current study, we showed that OmpA-sal remarkably stimulates T cell proliferation and IFN-γ production, which is a key cytokine of Th1 polarization through the increase in IL-12 production by DCs. These findings indicate that OmpA-sal from *S. enterica *serovar Typhimurium can induce the Th1 immune response by DC maturation and IL-12 production.

We also provide evidence that OmpA-sal activates TLR signaling pathways in DCs. The recognition of antigen by TLRs leads to activation of MAPK pathways in DCs [24]. Therefore, the activation of MAPK by OmpA-sal is a possible mechanism underlying the increased expression of IL-12 by DCs. In this study, we found that OmpA-sal binds to a TLR4 on DCs and activates MAPK signaling pathway-mediated IL-12 production. DC produces IL-12 and direct activation of CD4-positive T cells to differentiate into Th1 cells that produce high levels of IFN-γ [25,26]. We confirmed these results using TLR2^-/- ^DCs and TLR4^-/- ^DCs. OmpA-sal treated TLR2^-/- ^DCs or TLR4^-/- ^DCs and then analyzed IL-12 production by ELISA. We found that OmpA-sal-treated TLR4-/- DCs had no IL-12 production. These results suggest that OmpA-sal induced the maturation and activation of DCs via a TLR4-mediated signaling pathway.

## Conclusions

We demonstrated that OmpA-sal is a potent antigen and initiates a specific Th1 immune response *in vitro*. Further understanding of the mechanism by which OmpA-sal activates DC maturation and activation may facilitate the development of effective *S. enterica *serovar Typhimurim vaccines and an effective immunotherapeutic adjuvant for other infectious diseases.

## Methods

### Animals

Male 6-8 week old C57BL/6 (H-2K^b ^and I-A^b^) and BALB/c (H-2K^d ^and I-A^d^) mice were purchased from the Korean Institute of Chemistry Technology (Daejeon, Korea).

### Reagents and Antibodies

Recombinant mouse (rm)GM-CSF and rmIL-4 were purchased from R&D Systems. Dextran-FITC and LPS (from *Escherichia coli *055:B5) were obtained from Sigma-Aldrich. An endotoxin filter (END-X) and an endotoxin removal resin (END-X B15) were acquired from Associates of Cape Cod. Cytokine ELISA kits for murine IL-12 p70, IL-4, IL-10, and IFN-γ were purchased from BD Pharmingen. FITC- or PE-conjugated monoclonal antibodies (mAbs; BD Pharmingen) were used for flow cytometry to detect CD11c (HL3), CD80 (16-10A1), CD86 (GL1), IA^b ^β-chain (AF-120.1), H2K^b ^(AF6-88.5), IL-12 p40/p70 (C15.6), and IL-10 (JESS-16E3). Anti-phospho-ERK1/2, anti-phospho-p38 MAPK, anti-phospho-JNK1/2, anti-ERK1/2, anti-JNK1, and anti-p38 MAPK mAb were purchased from Cell signaling. Isotype-matched control mAbs and biotinylated anti-CD11c (N418) mAb were purchased from BD Pharmingen.

### Preparation of OmpA-sal

The full-length OmpA-sal gene (X02006.1) was amplified by PCR, and a chromosomal preparation of X02006.1 was used as a PCR substrate. The upstream primer, 5'-GCGGATCCCACGA AGCCGGAGAA-3', was designed to carry the EcoRI restriction site. The downstream primer, 5'-GCAAGCTTAGAAACGATAGCC-3', carried the HindIII restriction site. PCR products digested with EcoRI and HindIII were ligated into the pMAL™ expression vector (New England Biolabs Inc.). *E. coli *BL21 (DE3)/pMAL™ harboring a ompA-Sal gene was grown in Luria-Bertani (LB) medium at 37°C. Recombinant proteins were over-expressed by a bacteria protein expression system [27]. The quantity of OmpA endotoxin was ≤0.01 ng/mg.

### Generation and culture of DCs

DCs were generated from murine whole bone marrow (BM) cells. Briefly, the BM was flushed from the tibiae and femurs of BALB/c mice and depleted of red blood cells with ammonium chloride. The cells were plated in 6-well culture plates (10^6 ^cells/ml) and cultured at 37°C in 5% CO_2 _and OptiMEM (Invitrogen Life Technologies) supplemented with 10% heat-inactivated fetal bovine serum (FBS), 2 mM L-glutamine, 100 U/ml penicillin, 100 μg/ml streptomycin, 5 × 10^-5 ^M β-mercaptoethanol, 10 mM HEPES (pH 7.4), 20 ng/ml rmGM-CSF, and rmIL-4. On day 3 of culture, floating cells were gently removed and fresh medium was added. On day 6 or 7 of culture, non-adherent cells and loosely adherent proliferating DC aggregates were harvested for analysis or stimulation, or in some experiments, replated into 60 mm dishes.

### Quantitation of antigen uptake

In brief, DCs were equilibrated at 37°C or 4°C for 45 min, then pulsed with fluorescein-conjugated dextran at a concentration of 1 mg/ml. Cold staining buffer was added to stop the reaction. The cells were washed three times and stained with PE-conjugated anti-CD11c Abs, then analyzed with the FACSCalibur. Non-specific binding of dextran to DCs was determined by incubation of DCs with FITC-conjugated dextran at 4°C and subtracted as background. The medium used in the cultures with OmpA-sal stimulation was supplemented with GM-CSF, which is required for the ability of DCs to capture antigen.

### Cytokine assays

Cells were first blocked with 10% (v/v) normal goat serum for 15 min at 4°C, then stained with FITC-conjugated CD11c^+ ^antibody for 30 min at 4°C. Cells stained with the appropriate isotype-matched Ig were used as negative controls. The cells were fixed and permeabilized with the Cytofix/Cytoperm kit (PharMingen) according to the manufacturer's instructions. Intracellular IL-12p40/p70 and IL-10 were detected with fluorescein PE-conjugated antibodies (PharMingen) in a permeation buffer. The presence of murine IL-12p70, IL-10, IL-4, and IFN-γ in DCs was measured using an ELISA kit (R&D systems) according to the manufacturer's instructions.

### Cytoplasmic extracts and Western blot

The cells were exposed to LPS (200 ng/ml) with or without OmpA-sal pre-treatment (400 ng/ml). Following 5, 10, 15, or 30 min of incubation at 37°C, cells were washed twice with cold PBS and lysed with modified RIPA buffer for 15 min at 4°C. The protein content of cell lysates was determined using the Micro BCA assay kit (Pierce, Rockford, IL, USA). Equivalent amounts of proteins were separated by 10% or 12% SDS-PAGE and analyzed by Western blotting using anti-phospho-ERK1/2, anti-phospho-p38 MAPK, anti-phospho-JNK1/2, anti-ERK1/2, anti-JNK1, and anti-p38 MAPK mAb for 3 h, as described by the manufacturers.

### Mixed lymphocyte reaction

Responder T cells, which participate in allogeneic T-cell reactions, were isolated from spleens of BALB/c mice using a MACS column (positive selection sorting). Staining with FITC-conjugated anti-CD4 Abs revealed that the recovered cells consisted mainly of CD4^+ ^cells. The lymphocyte population was then washed twice in PBS and labeled with CFSE, as previously described [28]. The cells were washed once in pure FBS and twice in PBS with 10% FBS. DCs (1×10^4^), or DCs exposed to OmpA-sal or LPS for 24 h, were co-cultured with 1×10^5 ^allogeneic CFSE-labeled T lymphocytes in 96-well U-bottom plates. After 3 days, the CFSE dilution optically-gated lymphocytes were assessed.

### Evaluation of gene expression by real time PCR

TLR 2 and 4 PCR primers were used. Quantitative amounts of each gene were standardized against the GAPDH housekeeping gene. Real-time PCR was performed using a BioRad MiniOpticon System (BioRad Laboratories, Ltd.) with a SYBR green fluorophore. Reactions were performed in a total volume of 20 μl, including 10 μl of 2x SYBR Green PCR Master Mix, 1 μl of each primer at 10 ng, and 1 μl of the previously reverse-transcribed cDNA template. The protocols used were as follows: denaturation (95°C for 10 min), and amplification repeated 40 times (95°C for 30 s, 52°C for 30 s, 72°C for 30 s, and acquisition temperature for 15 s).

### Statistical analysis

All data are expressed as the mean ± standard deviation (SD) and were representative of at least two different experiments. Comparisons between individual data points were made using the Student's *t*-test and performed using one-way ANOVA analysis (Least Significant Difference (LSD) as post-hoc test). Throughout the figures and legends, the following terminology was used to denote statistical significance:**, *p *< 0.01, *, *p *< 0.05.

## Authors' contributions

Contribution: JSL performed research, analyzed data and wrote the paper; DJ and CML, JWP, and SHC performed research; TKH performed statistical analysis: SKJ, YKS and DJ K analyzed and interpreted data; JSL and YMP designed research, interpreted data and wrote the paper. All authors read and approved the final manuscript.
